# Millisecond mix-and-quench crystallography (MMQX) enables time-resolved studies of PEPCK with remote data collection

**DOI:** 10.1107/S2052252521007053

**Published:** 2021-08-04

**Authors:** Jonathan A. Clinger, David W. Moreau, Matthew J. McLeod, Todd Holyoak, Robert E. Thorne

**Affiliations:** a Physics Department, Cornell University, 142 Sciences Drive, Ithaca, NY 14853, USA; b Department of Biology, University of Waterloo, Waterloo, ON N2L 3G1, Canada

**Keywords:** time-resolved crystallography, enzymology, protein structure, structure determination, X-ray crystallography, enzyme mechanisms, millisecond mix-and-quench crystallography, protein dynamics, structural biology

## Abstract

Time-resolved crystallography of biomolecules in action has advanced rapidly as methods for serial crystallography have improved, but the large number of crystals and the complex experimental infrastructure that are required remain serious obstacles to its widespread application. Here, millisecond mix-and-quench crystallography (MMQX) has been developed, which yields millisecond time-resolved data using far fewer crystals and routine remote synchrotron data collection. To demonstrate the capabilities of MMQX, the conversion of oxaloacetic acid to phosphoenolpyruvate by phosphoenolpyruvate carboxy­kinase (PEPCK) is observed with a time resolution of 40 ms. By lowering the entry barrier to time-resolved crystallography, MMQX should enable a broad expansion in structural studies of protein dynamics.

## Abstract 2

Improvements in cryo-trapping technology enable single-crystal time-resolved crystallography of an enzyme with 40 ms time resolution.

## 1. Introduction   

Extending X-ray crystallography to yield ensemble-averaged all-atom, atomic resolution molecular movies of enzymes in action has long been a major goal in structural biology. Early time-resolved crystallography (TRX) experiments examined reactions that reset within the crystal and were triggered optically, such as CO release by myoglobin and the photocycle of photoactive yellow protein (PYP) (Schotte *et al.*, 2003 ▸; Kort *et al.*, 2004 ▸). These features allowed complete data sets at multiple time points to be obtained using a small number of crystals, with radiation damage limiting the amount of data collected from each crystal. The development of X-ray free-electron lasers (XFELs), with ultrabright femtosecond X-ray pulses that enable the capture of single-frame, fixed crystal orientation snapshots before most damage occurs, and of serial crystallography, enabling the delivery of tens of thousands of randomly oriented microcrystals into the XFEL beam and analysis of the resulting diffraction data sets, have driven major advances in TRX methods (Boutet *et al.*, 2012 ▸; Calvey *et al.*, 2016 ▸; Stagno *et al.*, 2017 ▸; Olmos *et al.*, 2018 ▸; Dasgupta *et al.*, 2019 ▸). These methods have been adapted for microfocus synchrotron sources (Monteiro *et al.*, 2020 ▸; Beyerlein *et al.*, 2017 ▸; Schulz *et al.*, 2018 ▸; Mehrabi *et al.*, 2019 ▸). Microcrystals enable chemical triggering via diffusion on millisecond timescales, which are short enough to reveal biologically important intermediate states.

Optical excitation and/or crystal mixing and delivery systems are complex and must be integrated into the X-ray beamline for TRX experiments. Optimization for a given protein crystal system and efficient serial data collection requires multiple collaborators, knowledgeable beamline staff and often extended beamline access. Complex sample-delivery systems, large sample consumption and limited access to suitably configured beamlines are formidable obstacles to the broad application of TRX techniques.

Freeze-quenching techniques provide an alternative approach to obtaining time-resolved data that has been productively used in spectroscopic studies (for example EPR) of liquid and powder samples (Tanaka *et al.*, 2003 ▸; Matsumura & Moënne-Loccoz, 2014 ▸) and in single-particle cryo-electron microscopy (Berriman & Unwin, 1994 ▸; Walker *et al.*, 1995 ▸; White *et al.*, 2003 ▸; Frank, 2017 ▸; Kontziampasis *et al.*, 2019 ▸; Yoder *et al.*, 2020 ▸; Mäeots *et al.*, 2020 ▸; Dandey *et al.*, 2020 ▸), including the analysis of ribosomes to observe conformational changes during elongation factor binding (Chen *et al.*, 2015 ▸; Fu *et al.*, 2016 ▸; Kaledhonkar *et al.*, 2019 ▸). Unlike in time-resolved studies conducted at room/biological temperature, freeze-quenching allows the reaction and measurement times to be separated, and long measurement times allow more data to be collected per sample per time point. Furthermore, in crystallography the radiation damage per unit dose is dramatically reduced at cryogenic temperatures, allowing more data to be collected per crystal.

Freeze-quenching was not considered to be viable for TRX studies, largely because the reactant-diffusion and crystal-cooling times for crystals large enough to yield adequate diffraction were longer than most timescales of biological interest (Teng & Moffat, 1998 ▸; Kriminski *et al.*, 2003 ▸; Warkentin *et al.*, 2006 ▸; Orville, 2018 ▸). However, the high brilliance of both XFELs and current-generation storage-ring light sources now enable data collection from micrometre-size crystals, for which diffusion times can be below 5 ms (Supplementary Table S1). Advances in liquid-nitrogen (LN_2_) cryocooling technology based on high-speed plunging and the removal of cold gas layers allow even ∼30 µm samples to be cooled to below 150 K in <3 ms (Warkentin *et al.*, 2006 ▸) and the vitrification of cryo-EM samples (Engstrom *et al.*, 2021 ▸). Diffusion and cooling times on these scales should allow the observation of slow dynamic processes and long-lived chemical intermediates, and the kinetic capture of main-chain and many side-chain conformations at biological temperature for low-temperature study.

We have developed a technique for mix-and-quench time-resolved crystallography by introducing substrate into microcrystals during a robotic plunge into LN_2_. This technique generates millisecond time-resolved structures using one or a few crystals per time point in a single shift of standard remote synchrotron data collection. MMQX thus promises to make TRX data collection much more broadly accessible.

## 2. Methods   

### 2.1. Plunge-cooler design   

**Figure 1:**
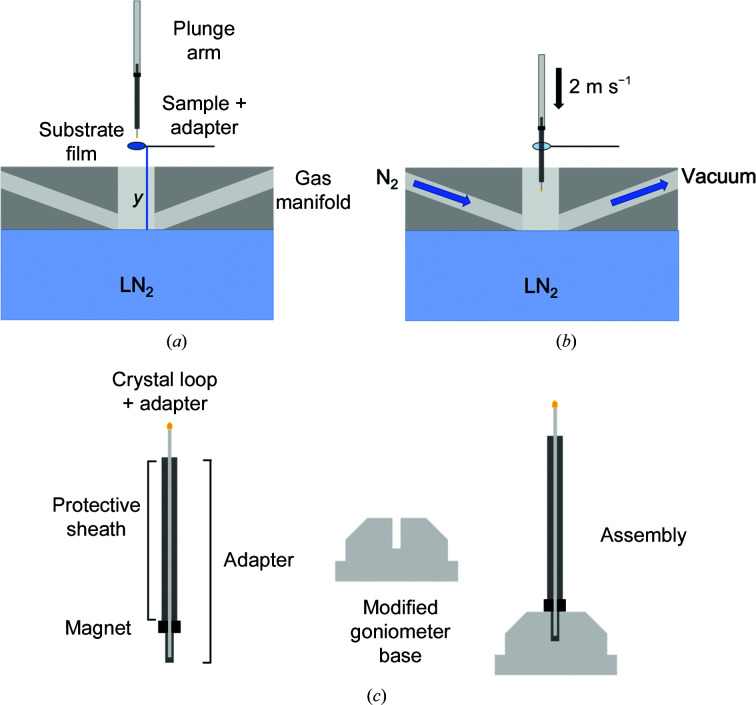
(*a*) Plunge-cooler design for MMQX, including a substrate-containing film. A polymer-film crystal mount on a stainless-steel pin in a magnetic holder is placed on a plunge arm attached to a high-speed linear-translation stage. To remove cold N_2_ gas that forms above the LN_2_ and ensure that the reaction proceeds at room temperature to the LN_2_ surface, a gas-management manifold flows warm N_2_ gas across the LN_2_ surface within its plunge bore, and removes cold and warm gas using vacuum. The time point *t* is determined by the height (*y*) of the film above LN_2_ and the plunge speed. (*b*) A crystal is plunged through the substrate film at a speed of 2 m s^−1^, then through room-temperature N_2_ gas present in the bore of the gas-management manifold to the LN_2_ surface and then through the LN_2_. (*c*) Pin adapter for plunge-cooling and MMQX experiments. To reduce the needed aperture size of the substrate-containing loop, the crystal is plunged in the pin holder. Once cooled, the pin holder is inserted into a modified ALS-format goniometer base. An optional protective sheath for pre-mixing incubations is shown.

The key requirements for LN_2_-based plunge-coolers to achieve the fastest possible cooling rates of small (sub-0.1 µl/500 µm) samples are (i) to plunge the sample at high speed into the LN_2_ and (ii) to eliminate sample precooling in cold gas normally present above the LN_2_ by carefully and thoroughly removing it (Warkentin *et al.*, 2006 ▸). As shown schematically in Fig. 1 ▸ and Supplementary Fig. S1, the plunge-cooler used here, based on a prototype design provided by MiTeGen LLC in 2015 that was then heavily modified for our purposes, consists of a vertical translation stage for the sample, a gas-management manifold immediately above the LN_2_ with a central bore coaxial with the plunge path, and an insulated chamber containing the LN_2_. The vertical translation stage has a carriage to which the plunge arm is attached, and this carriage moves up and down on a lead screw driven by a DC motor. The carriage accelerates from rest to a maximum speed of 2 m s^−1^, which it then maintains until it reaches a soft stop at a position where the sample is several centimetres below the LN_2_ surface, ensuring that the sample has cooled completely. A simple gravity-drop vertical stage (with, for example, electromagnetic sample release for precise timing) could also be used, but a motorized drive gives more flexibility to modulate the plunge speed to the LN_2_ and thus the time interval between sample mixing and cooling.

The gas-management manifold located directly above the LN_2_ has ports for dry room-temperature N_2_ gas and for suction provided by a Venturi vacuum generator. Heaters and insulation keep the manifold surfaces above the LN_2_ near room temperature. Prior to plunging, cold gas that forms above the LN_2_ is removed using warm dry N_2_ gas that is projected in a laminar flow across the LN_2_ surface and is removed via the vacuum port. As shown in Supplementary Fig. S2(*a*), with this cold gas-removal system and other design features of the manifold, the temperature remains above 273 K to within ∼100 µm of the LN_2_ surface. At 2 m s^−1^, the time to traverse the cold gas layer is then ∼50 µs, much less than sample-mixing times and sample-cooling times within the LN_2_ for all but the smallest crystals. Additional details of the plunge-cooler design are given in the Supporting Information.

Supplementary Fig. S2(*b*) shows temperature versus time during a plunge into LN_2_ recorded using a thermocouple with a bead roughly 100 µm in diameter and 50 µm thick. The time for the thermocouple to cool from the freezing point, *T*_f_ = 273 K, of pure bulk water to the protein–solvent glass transition (Ringe & Petsko, 2003 ▸; Doster, 2010 ▸) near 200 K is ∼3.5 ms, and that to the nominal glass-transition temperature of pure water, *T*_g_ ≃ 136 K, is ∼6 ms. These time intervals correspond to average cooling rates of ∼20 000 K s^−1^. Thermocouple metals have comparable heat capacity per unit volume between 273 and 200 or 136 K to protein crystals (including the latent heat of fusion of internal water; Thorne, 2020 ▸). For crystals in the sub-100 µm size range, cooling rates are determined by the thermal boundary layer in the surrounding N_2_, not the thermal conductivity of the sample (Kriminski *et al.*, 2003 ▸). Consequently, thermocouples and protein crystals of similar size should cool at similar rates, and the cooling rates should vary roughly as *L* ^−3/2^, where *L* is the linear sample size including both crystal and surrounding liquid. For the rod-shaped PEPCK crystals used here with transverse dimensions of 8 × 20 µm, visual observation of frozen samples suggested a thickness of crystal plus (substrate and PEG-containing) solution of roughly 20 µm, so cooling rates were likely to be >6 × 10^4^ K s^−1^ (Kriminski *et al.*, 2003 ▸) and cooling times from 273 to 200 K were <1.2 ms. These cooling times are sufficiently short to effectively trap room-temperature conformations of larger loops and flaps that undergo significant conformational changes when cooled at more typical rates in cryo-crystallography of <10^3^ K s^−1^ (Halle, 2004 ▸). With crystals a few micrometres in size and total crystal plus liquid thicknesses of ∼10 µm, cooling rates of ∼2 × 10^5^ K s^−1^ and cooling times of ∼0.5 ms should be achievable, which are within a factor of ∼2 of those achieved in cryo-EM practice using ethane: see, for example, Costello (2006 ▸) and Ravelli *et al.* (2020 ▸); see also Luyet & Gonzales (1951 ▸) for plunge-cooling measurements in liquid isopentane.

### 2.2. Sample preparation   

To test this approach to time-resolved crystallography, we used crystals of rat cytosolic phosphoenolpyruvate carboxy­kinase (PEPCK), an essential enzyme in glucose metabolism that catalyzes the reversible, nucleotide-dependent interconversion of oxaloacetic acid (OAA) and phosphoenol­pyruvate (PEP). PEPCK utilizes two divalent metal cations, M1 and M2, in its chemical activity, where M1 acts as a metal cofactor and M2 associates with the nucleotide in forming the substrate metal–nucleotide complex (Holyoak *et al.*, 2006 ▸). The kinetics and mechanism of this reaction are well understood, starting with OAA decarboxylation, releasing CO_2_ to form an enolpyruvate intermediate. This enolate intermediate then attacks the γ-phosphate of the GTP–M2 complex, producing PEP (Sullivan & Holyoak, 2008 ▸). Motions of various loops are required to orient the substrates to allow turnover. Most important is an Ω-loop that folds over the active site, protecting the enolate species from unwanted solvent protonation. This lid must reopen after phosphoryl transfer to allow product release (Sullivan & Holyoak, 2008 ▸). Structures obtained by co-crystallization and soaking have examined the binding of ligands including GTP, GDP, Mn^2+^, OAA and PEP, as well as stereochemical analogs of OAA, PEP and the enolate intermediate. Previous PEP-containing structures deposited in the PDB were obtained either using GDP-free crystals or using inactive mutants to prevent turnover. In these structures PEP is not found in the catalytically competent phosphate-transfer site but at a secondary site referred to as the outer-shell product-binding pocket, which is thought to be the location of PEP prior to release [Supplementary Fig. S3(*e*)]. However, phosphoglycolic acid, a mimic of PEP, has been observed in complex with GDP–M2, occupying the proposed catalytically active conformation where it cradles the M1 cofactor and its phosphate is in the γ-phosphate pocket, which is thought to be the productive substrate site (PDB entry 3dtb; Sullivan & Holyoak, 2008 ▸).

Mn^2+^ GTP–PEPCK co-crystals were prepared as described previously (Johnson & Holyoak, 2012 ▸). Briefly, rat cytosolic PEPCK protein was expressed, purified and concentrated to 10 mg ml^−1^ for crystallization experiments. PEPCK solution containing 20 m*M* MnCl_2_ and 5 m*M* GTP (both added immediately prior to crystallization) was mixed in a 1 µl:1 µl ratio in sitting-drop crystallization plates with reservoir solution consisting of 100 m*M* HEPES pH 7.5. 15–20%(*w*/*v*) PEG 3350. Suitable crystals were recovered from wells containing 19%(*w*/*v*) PEG 3350. The crystals used were rod-like, with dimensions of 100 × 20 × 8 µm. For crystals of this size, Δ*t*_cool_ was conservatively estimated to be 8 ± 4 ms and Δ*t*_diffuse_ was ∼12 ms (Supplementary Table S1).

### 2.3. Mixing experiment   

**Figure 2:**
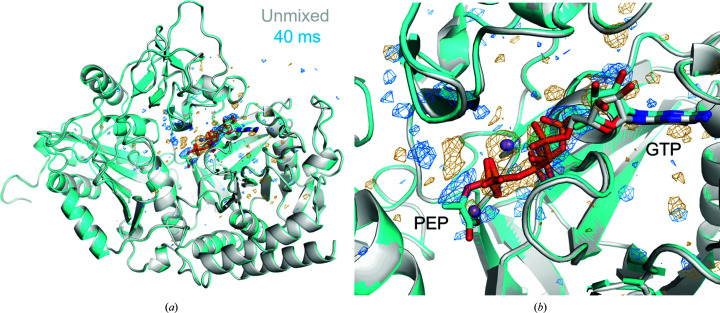
MMQX experiments show evidence for the diffusion of OAA into the PEPCK active site and formation of the PEP product. *F*_o_ − *F*_o_ analysis of 40 ms (cyan model) − unmixed (gray model) PEPCK data. *F*_o_ − *F*_o_ maps shown at ±3.5 r.m.s.d. (blue, orange). (*a*) Overview of the asymmetric unit demonstrating that large difference-map peaks are concentrated in the active site. (*b*) View of the active site, showing difference-map peaks for PEP and relaxation of GDP.

To initiate biochemical reactions in crystals, and to demonstrate the feasibility of collecting complete millisecond time-resolved structural data sets using as few crystals as possible and conventional cryo-crystallographic methods, we developed a plunge-through-film approach (Fig. 1 ▸). Individual PEPCK crystals were supported during plunging and data collection on MiTeGen MicroGrippers, which have narrow fingers projecting radially inwards from an outer loop that support the crystal while maximizing unobstructed access to the crystal surface. Crystal harvesting and placement on the sample carriage of the plunge-cooler was performed in an ∼85% relative humidity (r.h.) environment to minimize crystal dehydration between harvesting and plunging. As shown in Figs. 1 ▸(*a*) and 1 ▸(*b*), a thin substrate-containing film spanning a circular wire loop was placed along the plunge path of the crystal at a measured height above the LN_2_ surface. The stainless-steel pin of the MicroGripper was inserted into a cylindrical pin adapter of a custom two-part goniometer base [Fig. 1 ▸(*c*)]; this part had a diameter sized to pass through the film-containing loop and was attached to the sample arm on the sample carriage with a stainless-steel rod of a similar diameter. Crystals were blotted to remove excess liquid (essential to minimizing ligand-diffusion times into the crystal) and then plunged at 2 m s^−1^ through the film, which then collapsed around all sides of the crystal and the MicroGripper sample holder. The film was positioned at varying heights above the LN_2_ surface, creating different delays between the start of substrate diffusion into the crystal and the start of cryocooling. Critical to the success and interpretation of these experiments, the cold gas layer that would normally be present to a height of 2–6 cm above the LN_2_ surface was carefully removed (Fig. 1 ▸ and Supplementary Fig. S1), giving a transition from *T* > 273 K N_2_ gas to 77 K LN_2_ over a distance of ∼0.1 mm (Fig. 2 ▸) and ensuring that substrate diffusion and reaction occurred at room temperature until abrupt quenching on entering the LN_2_. After cooling/quenching, the sample and the pin adapter were removed from the sample arm of the plunge-cooler and inserted into part 2 of the goniometer base. The assembly was then loaded into UniPucks for subsequent data collection. This single-crystal plunge-through-film approach was chosen for its simplicity and robustness. Some applications of the mix-and-quench method may prefer to use more and smaller crystals, where simple microfluidic sprayers may be more appropriate than films.

The time point Δ*t* of the resulting mix-and-quench data set is estimated as Δ*t* = Δ*t*_plunge_ + Δ*t*_cool_ − Δ*t*_diffuse_, where Δ*t*_plunge_ = *v*_plunge_Δ*y* is the time for the crystal to plunge the distance Δ*y* between the substrate-containing film and the LN_2_ surface, Δ*t*_cool_ is the time for the sample to cool between room temperature and the protein–solvent glass transition near 200 K, and Δ*t*_diffuse_ is the time for the substrate to diffuse into the crystal, averaged over the X-ray illuminated crystal volume. With Δ*t*_cool_ = 8 ± 4 ms, Δ*t*_diffuse_ ≃ 12 ms (Section S6) and *v*_plunge_ ≃ 2 m s^−1^, Δ*y* values of 60 and 220 mm correspond to Δ*t*_plunge_ values of 30 ± 5 and 110 ± 5 ms, respectively. Time points are labeled using Δ*t*′ = Δ*t*_plunge_ + Δ*t*_cool_, with nominal values of 40 and 120 ms, respectively. The smallest feasible distance Δ*y* (limited by radiative cooling of the substrate-containing film, which can be minimized by translating the film into place immediately before the crystal plunge) is approximately 4 mm. Plunge speeds are limited to ∼5 m s^−1^ by cryogen splashing and sample-support damage, and these give a lower bound on Δ*t*_plunge_ of ∼0.8 ms.

### 2.4. Data collection and processing   

For PEPCK, the films through which the crystals were plunged contained 20 m*M* OAA, 20%(*v*/*v*) PEG 400 and 3%(*w*/*v*) SDS. OAA was the substrate in the reaction, PEG 400 provided cryoprotection of solution transferred from the film to the crystal surface, and SDS increased the stability of the film. PEPCK diffraction data were collected on the CHESS FLEXX beamline (Beamline 7B2) using a CRL lens-focused 9 × 12 µm X-ray beam of 2 × 10^11^ photons s^−1^ at an energy of 11 keV. The sample temperature was maintained at 100 K using a N_2_ gas cryostream, and diffraction data were recorded using fine slicing on a PILATUS 6M detector. Raw diffraction frames were processed with *DIALS* (Winter *et al.*, 2018 ▸). Data scaling and merging were performed with *AIMLESS* (Evans & Murshudov, 2013 ▸). *Phenix.refine* (Adams *et al.*, 2010 ▸) and *Coot* (Emsley *et al.*, 2010 ▸) were used for model building and refinement. *F*_o_ − *F*_o_ maps were generated with the isomorphous difference-map utility in *Phenix* (Liebschner *et al.*, 2019 ▸) using the substrate-mixed data at a nominal Δ*t*′ value of 40 ms to the maximum resolution of these data of 2.07 Å and using the well refined unmixed model at 1.84 Å resolution for phase information. *F*_o_ − *F*_o_ maps contoured at ±3.5 r.m.s.d. are shown in Figs. 2 ▸ and 3 ▸. Polder OMIT maps (Liebschner *et al.*, 2017 ▸) for the 40 and 120 ms time points were calculated from each data set while omitting GTP in the unmixed data and either PEP, carbon dioxide or GDP in the mixed data. See Supplementary Table S2 for diffraction and refinement statistics.

## 3. Results   

**Figure 3:**
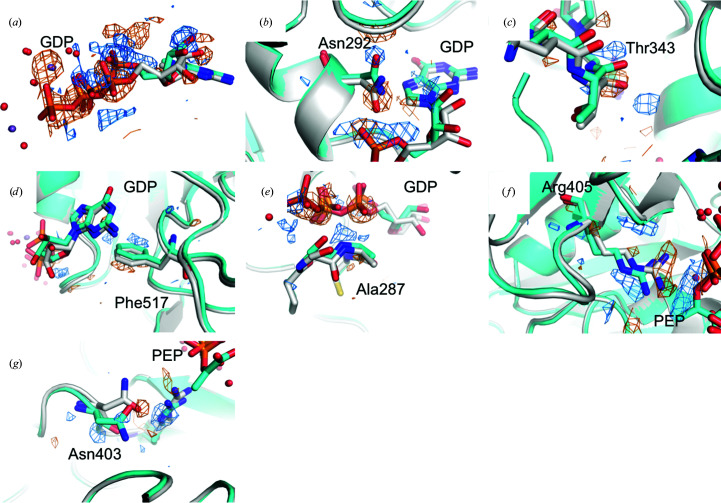
Side-chain and GTP-binding pocket motions associated with GDP relaxation 40 ms post-reaction. *F*_o_ − *F*_o_ maps are contoured at ±3.5 r.m.s.d. (positive, blue; negative, orange). (*a*) GTP (gray, umixed) − GDP (cyan, 40 ms) relaxation. (*b*) Asn292 motion. (*c*) Thr343 loop motion. (*d*) Phe517 side-chain breathing. (*e*) Ala287 motion into phosphate-binding cleft. (*f*) Arg405 motion away from the active site. (*g*) Asn403 twist away from the active site.

**Figure 4:**
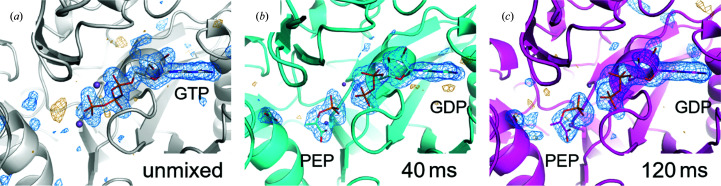
(*a*) Polder map of unmixed PEPCK (gray) data omitting GTP. (*b*) 40 ms time-point (cyan) Polder map omitting PEP, GDP and CO_2_. (*c*) 120 ms time-point (magenta) Polder map omitting PEP, GDP and CO_2_. All Polder maps are shown at 3.5 r.m.s.d..

**Figure 5:**
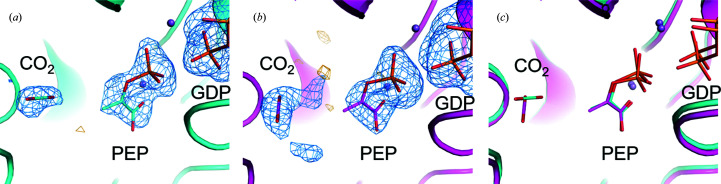
Comparison of CO_2_-binding sites at 40 and 120 ms and with a previous PEP-containing PEPCK structure. (*a*) Polder map at 4.5 r.m.s.d. of the CO_2_-binding site from the 40 ms MMQX data. (*b*) Polder map at 4.5 r.m.s.d. of the CO_2_-binding site from the 120 ms MMQX data. (*c*) Comparison of the CO_2_-binding sites at these two time points

Figs. 2–5 show electron-density maps obtained from diffraction data corresponding to delay times of Δ*t*′ = 40 and 120 ms after the start of mixing. Large changes in electron density compared with the apo structure and the features of these changes show that the substrate has entered the crystal and reaction has occurred.

In previous PEPCK structures obtained by standard ligand soaking and cryo-crystallographic data collection, the PEP product is observed in the product-release pocket (PDB ID 4gmw, Supplementary Fig. S3E). As shown in Figs. 3 ▸(*b*) and 4 ▸, in electron-density maps derived from diffraction data sets corresponding to Δ*t*′ ≃ 40 ms, we observe the PEP product in the phosphate-transfer site complexed by the Mn^2+^ active-site ion, similar to as in phosphoglycolic acid GDP–M2 complex, and not in the product-release pocket. This previously unobserved PEP position is the expected position immediately following phosphate transfer from GTP. The phosphate-to-phosphate distance of 3.4 Å compares with 7.5 Å in previous PEP+GDP structures. These results confirm previous hypotheses and fill in a gap in the structural dynamics of the reaction, in which the product is in the catalytically productive location instead of the release pocket where PEP has been previously observed (Supplementary Fig. S4). These early enzyme-product structural states help to understand the binding-pocket behavior during the PEPCK enzymatic reaction.

As the unmixed and Δ*t* ≃ 40 ms data are isomorphous, maps corresponding to *F*_o,mixed_ − *F*_o,unmixed_ can be calculated. These show clear hallmarks of GTP to GDP conversion, with relaxation of the α- and β-phosphates away from the Mn^2+^ catalytic ions [Figs. 2 ▸(*a*) and 2 ▸(*b*)]. The surrounding residues follow the GDP relaxation and breathe into the GDP-binding pocket (Fig. 3 ▸). As the Δ*t* ≃ 120 ms data are no longer isomorphous to the unmixed data, we cannot use as sensitive a method for analysis, but general inspection of the data suggests that little has changed in the active site between 40 and 120 ms. However, density further away from the active site is noticeably less well defined at 120 ms than in either the 40 ms or unmixed data, contributing to poorer refinement statistics. This may be due to crystal-to-crystal variation or to partially constrained motions involved in product release. The Ω-loop in all our structures is open and disordered, consistent with the hypothesis that the loop is open in unoccupied active sites as well as during the product-release steps after the reaction is catalyzed (Sullivan & Holyoak, 2008 ▸).

Polder maps calculated in the absence of any binding pocket ligands clearly show GTP in the unmixed data and PEP+GDP in the 40 and 120 ms data (Fig. 4 ▸). The phosphate transfer has clearly occurred and the distance between the β- and γ-phosphates is 3.4 Å, too far to model as covalently linked. There is a clear void in density between the phosphates, also indicative of GTP to GDP turnover. Furthermore, PEP is an excellent fit to the density, with real-space correlation coefficients (RSCCs) of 0.93 for 40 ms and 0.96 for 120 ms.

As shown in Fig. 5 ▸, our 40 and 120 ms post-mixing structures also exhibit density that can be tentatively attributed to CO_2_, which was formed from OAA during the generation of the enolpyruvate intermediate. The chemical identity of the carboxylating agent as well as its specific binding site have been contentious (Tang *et al.*, 2018 ▸; Cotelesage *et al.*, 2007 ▸), and these TRX data are consistent with prior kinetic data indicating that CO_2_ (rather than bicarbonate) is the form of the substrate for carboxylation of PEP (Cooper *et al.*, 1968 ▸). Relative to the 40 ms structure, our 120 ms structure indicates relaxation of the density attributed to CO_2_ to a different orientation in the binding pocket, where it makes favorable interactions with the main-chain N atoms of Arg87 and Gly237, as well as the side chain of Asn403. Real-space correlation coefficients of 0.89 in the 40 ms data and 0.87 in the 120 ms data indicate a high probability of the density being CO_2_, but with more uncertainty than our identification of PEP.

## 4. Discussion   

We have demonstrated the feasibility of millisecond mix-and-quench crystallography (MMQX) by using it to trap a previously unobserved structural state of an enzyme *in crystallo*. We obtained complete structural data sets at time points of 40 and 120 ms using a single crystal for each time point.

Data are collected at cryogenic temperatures, where the radiation-damage limits are 50 or more times larger than at room temperature (Warkentin *et al.*, 2017 ▸; Atakisi *et al.*, 2019 ▸; de la Mora *et al.*, 2020 ▸). Since the reactions are quenched, the data-collection times and the desired time resolution are decoupled, and neither is limited by the other. Consequently, far more data can be collected per crystal than is feasible in room-temperature serial crystallography approaches. Sample usage in MMQX is comparable to that in standard cryo-crystallo­graphy and is at least two orders of magnitude lower than in the most sample-efficient room-temperature synchrotron-based time-resolved methods.

The time resolution that we have achieved in MMQX is comparable to the best reported using serial mixing crystallography to date of 30 and 15 ms (Olmos *et al.*, 2018 ▸; Mehrabi *et al.*, 2019 ▸). Using crystals of a few micrometres in size to reduce diffusion times and cooling times and current LN_2_-based cooling technology, this method should yield time resolutions of a few milliseconds, comparable to those achievable using any synchrotron-based serial mixing technique and within an order of magnitude of solution-based techniques, including freeze-quench EPR and other freeze-quench spectroscopic methods.

The only cost of these benefits is the possible relaxation of some structural features during cooling from room temperature (Halle, 2004 ▸), such as alternate conformations of side-chain rotamers (Keedy *et al.*, 2014 ▸, 2015 ▸) and coordination of water molecules. However, larger structural features such as the positions of loops and flaps and coordinated products are likely to be effectively trapped by cooling times that can reach the submillisecond range. These cooling times are comparable to (and no more than a factor of 2–6 smaller than) those achieved in current cryo-EM practice (Engstrom *et al.*, 2021 ▸). While crystal contacts and constrained solvent spaces may inhibit some mechanistically important ligand-binding-induced conformational motions, they may also stabilize conformations against cooling-induced changes. Microcrystal-based MMQX and time-resolved cryo-EM may thus be comparable in the fidelity with which they capture configurations generated in reactions occurring at room/biological temperature.

While the plunge-through-film method for introducing substrate into crystals is convenient, it may not be suitable to achieve the smallest time resolutions and fastest cooling rates when using few micrometre-sized crystals. For these applications, samples on a support may be sprayed with substrate before or during plunging using, for example, a microfluidic sprayer, or microcrystals can be mixed and sprayed onto the support during the plunge, in much the same way as grids are prepared for time-resolved cryo-EM studies.

Using either the plunge-through-film or microfluidic sprayer approach to MMQX, millisecond time-resolved information can be obtained using mail-in remote data collection on standard cryo-crystallography synchrotron beamlines without modification and using crystals left over from initial structure determinations. Consequently, this method should expand access to the high-impact field of time-resolved crystallography and enable structural enzymology to become a powerful tool for many scientists. MMQX is complementary to time-resolved serial femtosecond crystallographic methods. Data collection under physiological conditions with negligible radiation damage is exchanged for orders-of-magnitude less sample consumption, much simpler sample preparation and much greater beamtime access for iteration and optimization.

## 5. Related literature   

The following references are cited in the supporting information for this article: Akinkunmi *et al.* (2015 ▸), Cvetkovic *et al.* (2005 ▸), Geremia *et al.* (2006 ▸), Holton (2009 ▸), Holton & Frankel (2010 ▸), Ribeiro *et al.* (2006 ▸), Sanishvili *et al.* (2011 ▸), Schmidt (2013 ▸), Tominaga & Matsumoto (1990 ▸) and Yamamoto *et al.* (2017 ▸).

## Supplementary Material

PDB reference: PEPCK, steady-state structure with Mn^2+^ and GTP, 7l36

PDB reference: MMQX structure 40 ms post-mixing with oxaloacetic acid, 7l3m

PDB reference: MMQX structure 120 ms post-mixing with oxaloacetic acid, 7l3v

Supporting Information including Supplementary Tables and Supplementary Figures. DOI: 10.1107/S2052252521007053/ro5030sup1.pdf
