# Supplementary material for: Millisecond mix-and-quench crystallography (MMQX) enables time-resolved studies of PEPCK with remote data collection
Source: IUCrJ. 2021 Aug 4;8(Pt 5):784–92. doi: 10.1107/S2052252521007053 (PMC8420759; doi:10.1107/S2052252521007053)
Supplement: Supplementary file 1 [file m-08-00784-sup1.pdf]

# IUCrJ

**Volume 8 (2021)**


**Supporting information for article:**

## Millisecond mix-and-quench crystallography (MMQX) enables time-resolved studies of PEPCK with remote data collection

**Jonathan A. Clinger, David W. Moreau, Matthew J. McLeod, Todd Holyoak and Robert E. Thorne**

#### S1. Design and fabrication of sample holders

Sample holders (Fig. 1(**C**)) are designed around ALS-style goniometer bases to facilitate automated handling at the synchrotron. The sample loop/MicroGripper's stainless steel rod slides into a hollow insert with a small magnet near one end, which is then inserted into a custom ALS-style magnetic steel base. Only the sample loop + insert is plunged through the substrate-containing film into the $LN_2$, allowing a smaller diameter, more stable film to be used. After plunge cooling, the insert is removed from the apparatus and placed into the goniometer base while being kept inside the $LN_2$ dewar. The insert consists of an electrical pin receptacle (Digikey: ED90590-ND, Mill-Max: 0677-0-15-80-30-27-10-0) that is soldered into a ring magnet (K&J Magnetics: R211). A standard crystal mount fits into the receptacle. Optionally, a thin metal sheathe can be soldered onto the magnet which supports MiTeGen MicroRT$^{TM}$ tubes and glass capillaries for room temperature data collection and pre-plunging incubations. The goniometer base is made from an ALS style base (MiTeGen: B1A). Figure 1 shows the insert cross-section with the optional sheathe attached. The top is machined flat and a bevelled through hole is made for the insert. The bevel is necessary to aid in inserting the insert, the through hole needs to be free from any burs.

#### S2. Design and fabrication of $LN_2$ Dewar

The $LN_2$ dewar was made from high density polyethylene foam. The 1" thick foam was sliced with a razor blade while the 4" blocks were cut with a band saw. The blocks were bonded together by briefly heating their surfaces with a heat gun, then pressing them together under force. Cryogenic epoxy (Stycast 2850FT) was applied to the joints as additional sealant. A Dewar lid was made from the 1" foam and fits around the cold gas removal apparatus.

#### S3. Gas management manifold design

Figure S1 shows a cross-section of the gas management manifold that rests on top of the Dewar and extends down to the $LN_2$ surface. The manifold has a plunge bore 1 cm in diameter, through which the sample passes to the $LN_2$, and includes a tube extending below the $LN_2$ surface. The tube isolates the gas present within the plunge bore from that present above the $LN_2$ in the rest of Dewar, allowing it to be separately managed, and reduces the amount of cold gas that must be removed. The lower portion of the manifold is machined from a low thermal conductivity glass-mica ceramic. The upper portion defining the $N_2$ gas and vacuum channels and the plunge bore is machined from Teflon, and is heated by two cartridge heaters to prevent

frosting. Vacuum is generated using a venturi vacuum generator (Gast: VG-015-00-00) and controlled by a solenoid (Asco 8262H022). This vacuum generator is only operated for a few seconds before the sample is plunged; otherwise, it draws in humid air, causing frosting. A second port provides a small amount of warm dry $N_2$ gas to prevent humid air from entering the plunge bore. An aluminum block forms the top of the manifold and is heated by a ring heater from above (Chromalox: 135263). A thermocouple (Omega: 5SC-TT-K-36-180) is located inside the manifold near the sample path and is connected to a PID temperature controller (Watlow 965) that sends power to the heaters.

### S4. Sample plunging mechanism design

The sample loop + insert attaches to a plunge arm, which in turn is attached to a carriage on a screw-driven linear translation stage. The screw is rotated by a DC motor that is controlled via a motor controller using Labview. A magnetic sensor at the top of the track is used for homing the translation stage. At the bottom of the stage, the carriage impacts padded aluminium stops that move on a separate rail and that compress a dashpot and a spring, bringing the carriage to a smooth stop.

### S5. Thermocouple measurements characterizing the performance of the plunge cooler

Figure S2(A) shows the temperature, measured using a type-K thermocouple having a bead roughly 100 μm in diameter and 50 μm thick, as a function of height $y$ above the surface of the $LN_2$ in the plunge cooler's plunge bore, as the thermocouple was slowly stepped toward the $LN_2$ surface. During the measurement, cold gas above the $LN_2$ within the plunge bore was removed by the gas management manifold using vacuum and make-up dry ambient temperature gas, and the bore walls were heated using cartridge heaters as described above. Figure S2(B) shows the temperature versus time measured for different plunge speeds when using the same thermocouple as in Fig. S2(A). The cooling time of the thermocouple bead is larger than is expected for the 100 μm × 20 μm × 8 μm crystals used here, as cooling times in both cases should be dominated by the smaller sample dimensions.

### S6. Estimates of diffusion times for OAA and glucose

Our estimates of diffusion times for OAA into PEPCK crystals and glucose into GI crystals are based on a solution to Fick's second law for a cuboid crystal described by Schmidt (Schmidt, 2013; Mehrabi *et al.*, 2019). The smallest dimension of the crystal dominates the diffusion time. Needle and plate morphology crystals are favourable in mixing experiments, as they

have larger diffracting volumes relative to their diffusion times. This analysis ignores the fact that the solvent is nanoconfined within channels of a protein crystal, that viscosity may be affected by this confinement, and that substrate may have weak binding interactions with the protein surface, all of which are likely to slow diffusion (Cvetkovic *et al.*, 2005; Geremia *et al.*, 2006). This analysis also does not account for any solvent present on the crystal surface prior to mixing, which could slow diffusion by providing additional liquid through which the substrate must diffuse and by reducing the substrate concentration at the crystal surface (Mehrabi *et al.*, 2019). Here, considerable care was taken to remove that surface solvent by blotting immediately prior to plunging.

No diffusion constant measurements for OAA are available. The diffusion constant of erythritol, which has a similar molecular weight and hydrodynamic radius to OAA, of $7.6 \times 10^{-6}$ $cm^2/s$ in dilute aqueous solution at 295 K(Tominaga & Matsumoto, 1990) is used. Glucose has a diffusion coefficient of $6.3 \times 10^{-6}$ $cm^2/s$ in dilute aqueous solution at 295 K (Ribeiro *et al.*, 2006).

Table S1 gives the resulting diffusion time estimates for OAA into PEPCK crystals and for glucose into glucose isomerase crystals, for crystals of different sizes. In addition to the limitations noted above, these estimates also ignore any precooling of the sample in cold gas as it descends from the substrate-containing loop to the $LN_2$ surface, which could dramatically increase diffusion times in small crystals. For example, the diffusion coefficient of glycerol at 273 K is ~40% of its value at 295 K (Akinkunmi *et al.*, 2015). This precooling is avoided in our plunge cooler design but is usually substantial when plunge cooling into $LN_2$ contained within foam or glass Dewars.

Diffusion time estimations do not affect the nominal time point in these or other mixing experiments, as the enzymatic reaction will occur in the exterior asymmetric units while diffusion into the center of the crystal is on-going. Instead, diffusion times are best described as the fastest timepoints where full occupancy is achievable, as well as the spread of observed time points in the crystal(s). As an example, the estimated diffusion time in our 100x20x8 $\mu m^3$ crystals is 12ms. The observed timepoint by each aysmetric unit in our nominal 40ms timepoint ranges from 40ms to 28ms assuming the estimated diffusion time holds.

## S7. Estimate of minimum number of crystals for mix-and-quench experiments required to obtain complete data sets at synchrotron sources

To estimate the crystal efficiency of MMQX relative to room temperature time-resolved SSX methods (Table S1), we used the required crystal number / size calculator developed by

Holton and Frankel at https://bl831.als.lbl.gov/xtalsize.html (Holton, 2009; Holton & Frankel, 2010). Our calculations make the following assumptions: the crystal is sized to achieve a desired mixing time, the X-ray beam size is matched to crystal size, the screening image diffracts to 1.5 Å, and the required merged resolution is 2.0 Å with an I/ σ at 2.0 Å of 1.4. These parameters are reasonable for our PEPCK MMQX data, as many of our crystals show diffraction peaks to 1.6 Å or higher, and a 2.0 Å cutoff is sufficient to obtain required map features. I/ σ of 1.4 is between the traditional 2.0 cut-off and the more aggressive merging utilized with $CC_{1/2}$ resolution cutoffs. Our calculations indicate that for PEPCK and glucose isomerase, one crystal should allow collection of a complete dataset to time points of 30 ms or slower. The number of crystals required increases with macromolecule/asymmetric unit size; for ribosomes at least 13 crystals would be required to collect data at a 30 ms time point for a diffusing ligand comparable in size to glucose. The small numbers of crystals required for MMQX compared with current TR serial crystallography methods allows collection of many more time points with a given sample amount, or experiments to be performed using multiple substrates/ligands. Ongoing advances in data collection procedures, e.g., utilizing X-ray beam offsets (Yamamoto *et al.*, 2017) or utilizing very hard X-rays (Sanishvili *et al.*, 2011) at microfocus synchrotron sources will make MMQX more sample efficient.

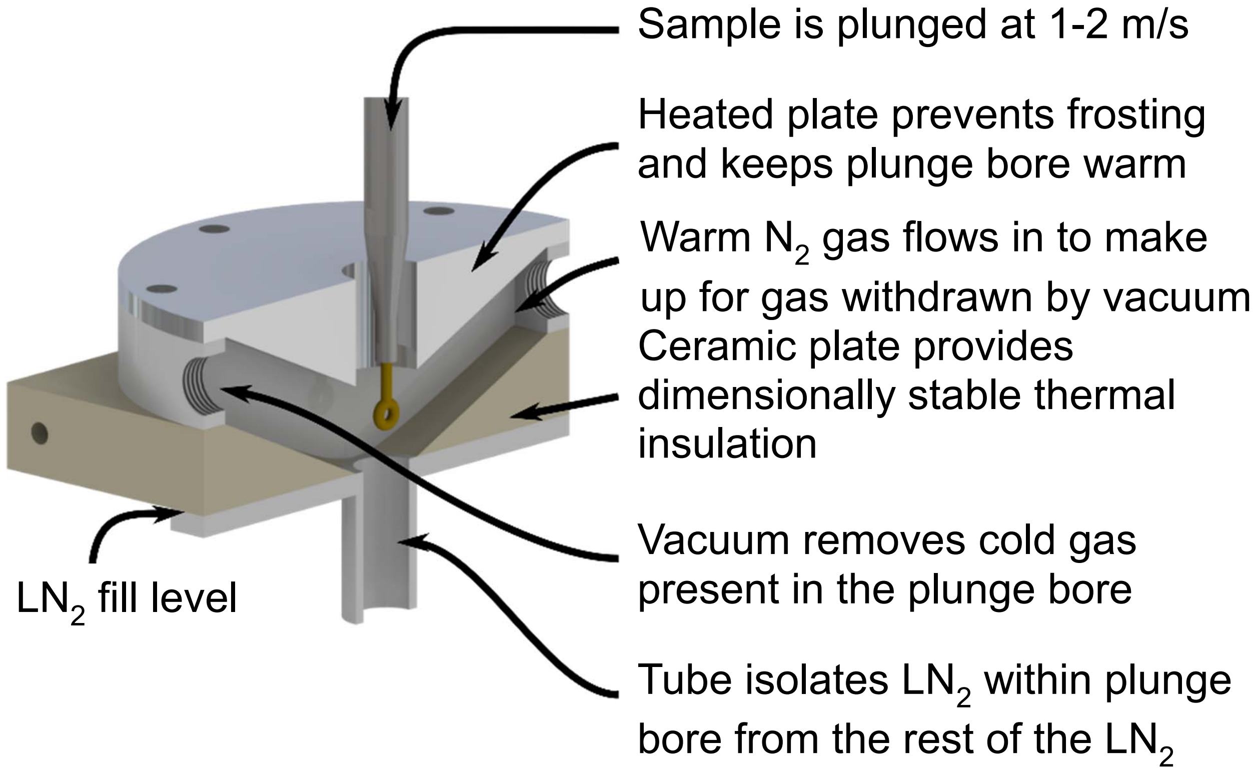


**Figure S1** Cross-section of the gas management manifold of the plunge cooler in Fig. 1.

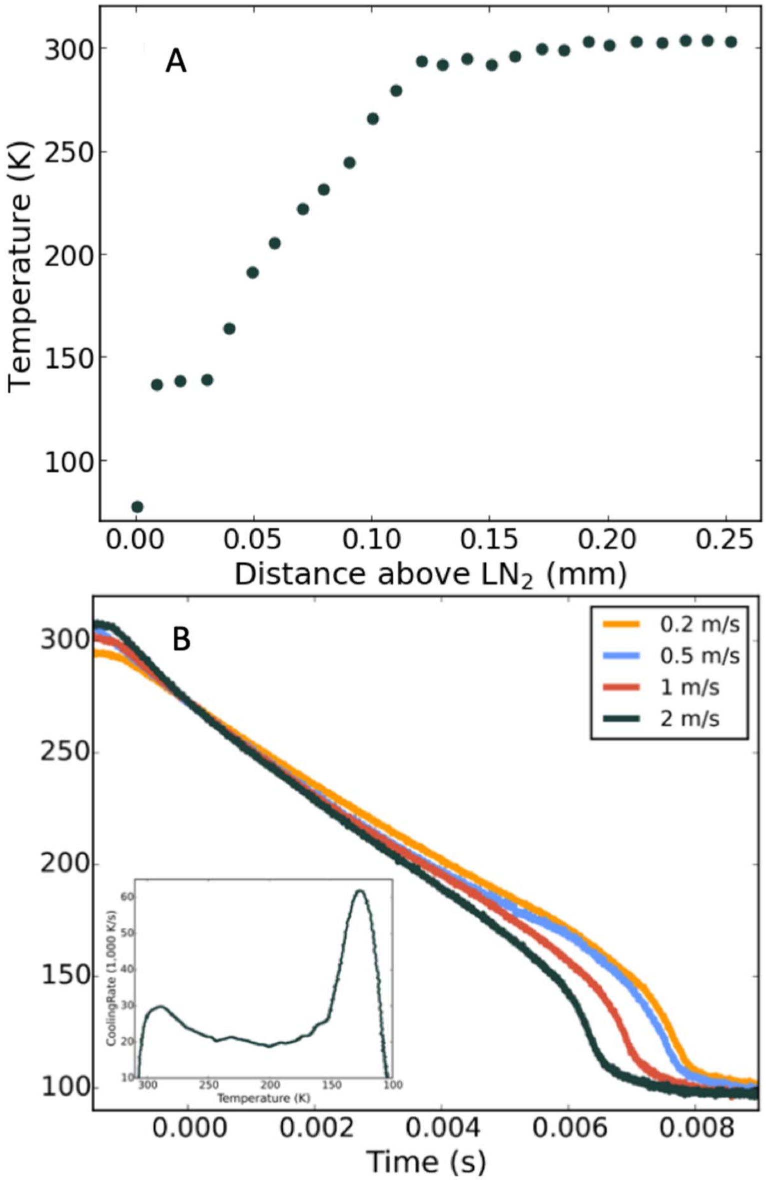


**Figure S2** Measurements, using a thermocouple with a junction bead roughly 100 μm in diameter and 50 μm thick, characterizing the performance of the plunge cooling system. **(A)** Temperature vs height *y* above the $LN_2$ surface along the sample plunge path, measured using a thermocouple held on the sample arm of the plunge cooler. With cold gas removal and replacement by the gas management manifold, the temperature remains above 273 K to within ~0.1 mm of the $LN_2$ surface. **(B)** Temperature vs time recorded during plunges at speeds between 0.2 and 2 m/s. The time $t$=0 is set by the time when the thermocouple temperature reaches 273 K, which occurs very close to (just above or below, depending on plunge speed) the $LN_2$ surface. For a plunge speed of 2 m/s, the cooling time from 273 K to 150 K is 5.8 ms. For samples the size of the PEPCK crystals used in our MMQX experiments (rods with a cross section of 20 μm × 8 μm), cooling times from room temperature to 150K should be less than 10 ms and likely on the order of 1-2 ms.

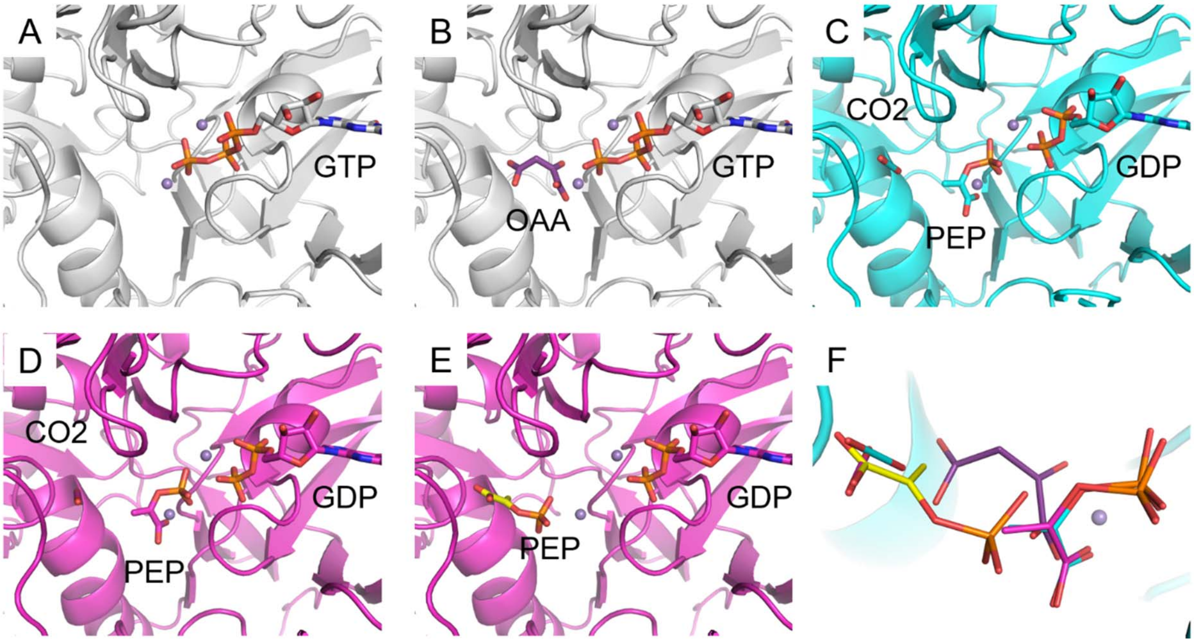


**Figure S3** PEPCK binding pocket structures and overview of reaction steps including the MMQX structures. **(A)** PEPCK Mn (purple spheres) and GTP binding pocket before substrate binding. **(B)** OAA binding location added from PDB ID 2QF1 (purple). **(C)** 40 ms timepoint binding pocket. PEP + GDP and possible CO2 release pocket (cyan). **(D)** 120 ms timepoint pocket (magenta). **(E)** PEP location in the release channel from PDB ID 4GMW (yellow) added into the 120 ms model. **(F)** Substrate and product release pocket alignment of all structures. OAA (purple) binds first and is then converted to PEP+$CO_2$ (40ms-cyan, 120ms magenta). Eventually PEP transitions from the catalytic site to the product release pocket (yellow).

**Table S1.** Estimates of diffusion times (Schmidt, 2013) and number of crystals required in order to collect a full dataset (Holton & Frankel, 2010; Holton, 2009), for protein crystals of different sizes.

| | PEPCK crystals | | Glucose Isomerase crystals | | Eukaryotic Ribosome crystals |
|---|---|---|---|---|---|
| Crystal size ($\mu m^3$) | OAA diffusion time (ms) | crystals per dataset | glucose diffusion time (ms) | crystals per dataset | crystals per dataset |
| 2×2×2 | 0.2 | 100 | 0.3 | 240 | 4300 |
| 5×5×5 | 1.1 | 12 | 1.7 | 28 | 490 |
| 10×10×10 | 4.5 | 2 | 6.8 | 4.9 | 85 |
| 10×20×30 | 9.8 | 0.45 | 14.9 | 1.1 | 19 |
| 20×20×20 | 17.8 | 0.31 | 27.1 | 0.75 | 13 |
| 30×30×30 | 40.0 | 0.1 | 60.9 | 0.24 | 4.2 |
| 40×40×40 | 71.2 | 0.04 | 108.2 | 0.11 | 1.8 |

**Table S2.** Data collection and refinement statistics.

| | Unmixed PEPCK + Mn & GDP | 40ms post OAA mixing PEPCK | 120ms post OAA mixing PEPCK |
|---|---|---|---|
| PDB ID | 7L36 | 7L3M | 7L3V |
| Wavelength (Å) | 1.127 | 1.127 | 1.127 |
| Resolution range (Å) | 41 - 1.84 (1.906 - 1.84) | 51.33-2.07 (2.14 - 2.07) | 41.67-1.98 (2.05-1.98) |
| Space group | P 1 21 1 | P 1 21 1 | P 1 21 1 |
| Unit cell (Å) | 44.38 118.55 60.23 90 109.53 90 | 44.43 118.9 60.37 90 109.5 90 | 46.15 118.81 61.23 90 107.3 90 |
| Total reflections | 470638 (16681) | 127784 (11833) | 156173 (14490) |
| Unique reflections | 50261 (4755) | 35866 (3525) | 43789 (4237) |
| Multiplicity | 9.4 (3.5) | 3.6 (3.4) | 3.6 (3.4) |
| Completeness (%) | 98.84 (93.6) | 99.6 (97.7) | 99.4 (96.3) |
| Mean I/sigma(I) | 8.74 (1.17) | 6.09 (2.18) | 5.45 (1.48) |
| Wilson B-factor | 15.3 | 18.7 | 23.9 |
| R-merge | 0.228 (1.30) | 0.166 (0.878) | 0.148 (1.14) |
| R-meas | 0.242 (1.53) | 0.196 (1.05) | 0.175 (1.37) |
| R-pim | 0.078 (0.79) | 0.109 (0.56) | 0.093 (0.74) |
| CC1/2 | 0.987 (0.38) | 0.967 (0.49) | 0.937 (0.52) |
| CC* | 0.997 (0.74) | 0.992 (0.81) | 0.984 (0.83) |
| Reflections used in refinement | 50263 (4727) | 35840 (3515) | 43715 (4215) |
| Reflections used for R-free | 2610 (245) | 1857 (199) | 2198 (237) |
| R-work | 0.152 (0.266) | 0.172 (0.268) | 0.186 (0.275) |
| R-free | 0.199 (0.299) | 0.186 (0.323) | 0.206 (0.318) |
| Number of non-hydrogen atoms | 5304 | 5234 | 5230 |
| macromolecules | 4840 | 4893 | 4780 |
| ligands | 36 | 45 | 45 |
| solvent | 428 | 324 | 433 |
| Protein residues | 613 | 612 | 601 |
| RMS(bonds) | 0.004 | 0.007 | 0.011 |
| RMS(angles) | 0.75 | 0.99 | 1.20 |
| Ramachandran favored (%) | 97.04 | 97.04 | 96.64 |
| Ramachandran allowed (%) | 2.96 | 2.80 | 3.36 |

| | | | |
|---|---|---|---|
| Ramachandran outliers (%) | 0.00 | 0.16 | 0.00 |
| Rotamer outliers (%) | 0.00 | 0.97 | 0.60 |
| Clashscore | 3.41 | 5.02 | 5.05 |
| Average B-factor | 22.5 | 23.7 | 42.4 |
| macromolecules | 21.9 | 23.2 | 41.7 |
| ligands | 18.8 | 42.4 | 57.8 |
| solvent | 29.0 | 29.3 | 49.7 |
| Ligand real-space correlation coefficient | | | |
| GTP | 0.98 | | |
| GDP | | 0.96 | 0.96 |
| PEP | | 0.93 | 0.96 |
| $CO_2$ | | 0.89 | 0.87 |

Statistics for the highest-resolution shell are shown in parentheses.
